# Navigation in reverse total shoulder arthroplasty: a retrospective study

**DOI:** 10.1016/j.xrrt.2026.100718

**Published:** 2026-03-06

**Authors:** Randall A. Arroyo, Sierra J. Casper, Christopher D. Joyce, Robert Z. Tashjian, Peter N. Chalmers

**Affiliations:** a University of Utah, Department of Orthopaedic Surgery, Salt Lake City, UT, USA; b University of California, School of Medicine, Riverside, CA, USA; c University of Utah, Spencer Fox Eccles School of Medicine, Salt Lake City, UT, USA

**Keywords:** Computer-assisted navigation, Learning curve, Reverse total shoulder arthroplasty, Pre-operative planning

## Abstract

**Background:**

The purpose of this study was to evaluate radiographic accuracy and assess the learning curve associated with a single surgeon's transition from non-navigated to navigated reverse total shoulder arthroplasty (rTSA).

**Methods:**

This was a retrospective clinical study that included 166 consecutive rTSA cases performed by a single surgeon, with 84 non-navigated cases immediately followed by 82 navigated rTSA using computer-assisted navigation. Radiographic measurements included pre- and post-operative β-angle (inclination), inferior glenosphere offset, and the distance from the center of rotation of the glenosphere to the tip of the coracoid (COR–C). For all radiographic measurements, intrarater reliability was compared using intraclass correlation coefficients. Statistical comparisons were performed using *t*-tests, Mann-Whitney U, and Fisher–Freeman–Halton tests.

**Results:**

All radiographic measurements had excellent reliability with intraclass correlation coefficients of >0.799. Inferior offset was significantly greater in the navigated group (7.0 ± 2.5 mm) than in the non-navigated group (5.4 ± 3.1 mm; *P* = .001). No differences were found in post-operative inclination or COR–C distance. Operative time was significantly higher with navigation (141 ± 23.5 min vs. 136 ± 30.8 min; *P* = .031). Early dislocations occurred in 4.7% of non-navigated cases and in 0% of navigated cases (*P* = .045).

**Conclusion:**

This retrospective single-surgeon study illustrates that transition to computer-assisted navigation in rTSA may modestly increase operative time while improving implant positioning including inferior glenoid offset. No early postoperative dislocations were observed in the navigated group, though definitive conclusions are limited by short follow-up and limited sample size. This study demonstrates an association between the role of computer-assisted navigation in improving implant accuracy, but further studies are needed to determine its clinical relevance.

Since its approval by the Food and Drug Administration in 2003, reverse total shoulder arthroplasty (rTSA) has been used as an effective treatment for a wide variety of indications including rotator cuff arthropathy, proximal humeral fractures, and for revisions of prior arthroplasty.^27^ rTSA has overall good outcomes, but many patients fail to achieve full internal and external rotation post-operatively, in part because of the increased constraint within the reverse.^3^^,^^8^ Range of motion correlates with patient-reported outcomes; therefore, the success of rTSA is heavily dependent on achieving an impingement-free arc to optimize range of motion.^1^ Therefore, accurate glenoid component positioning is critical to the success and long-term outcomes for patients undergoing rTSA, as poor positioning can contribute to instability and impingement.^15^^,^^25^

Glenoid component placement is technically challenging and critically important to the success of shoulder arthroplasty.^10^^,^^22^^,^^26^ Perfecting rTSA requires optimally positioning the arc of impingement-free motion within a patient's functional range and because this arc is determined in part by glenoid component, it is among the most important aspects of the rTSA. One possible solution to improving accuracy and achieving optimal positioning may be through the introduction of computer-assisted navigation.^17^ Unlike pre-operative planning alone, which relies on visual estimation by the surgeon, computer-assisted navigation allows for a direct translation of the pre-operative plan to the actual implant placement during surgery. This technology has become a rapidly popular tool for shoulder arthroplasty, with a recent 7-year period from 2017 to 2023 demonstrating a nearly 15-fold increase in navigated total arthroplasties.^28^ This technology has also demonstrated a reduction in cage perforation and an increase in glenoid screw length, which may improve baseplate stability.^21^

Despite the rapid integration of computer-assisted navigation in rTSA, the evidence of its efficacy remains limited. There are conflicting results regarding the cost-effectiveness of navigation and limited evidence on the impact on operative time, instability, and the associated learning curve utilizing the new technology.^23^ The current literature has demonstrated an improvement in implant accuracy while also reducing the differences in positioning between novice and experienced surgeons.^13^ While these findings are important for understanding the operative accuracy, they do not address operative efficiency or complications for a surgeon transitioning to navigation. Moreover, the data currently available is derived from heterogeneous, multisurgeon series or industry-sponsored registries.^12^^,^^29^ There remains a need for an independent, single-surgeon study to understand the true value of transitioning to navigation in rTSA.

The purpose of this study was to evaluate the learning curve associated with the adoption of navigation in rTSA by assessing its impact on operative time, radiographic implant accuracy, and early complication rates compared to non-navigated rTSA. We hypothesized that the implementation of navigation would increase operative time with a tradeoff of increasing implant positioning accuracy which subsequently could lead to reduced complication rates compared to the conventional, non-navigated group.

## Methods

### Patient selection

**Figure 1:**
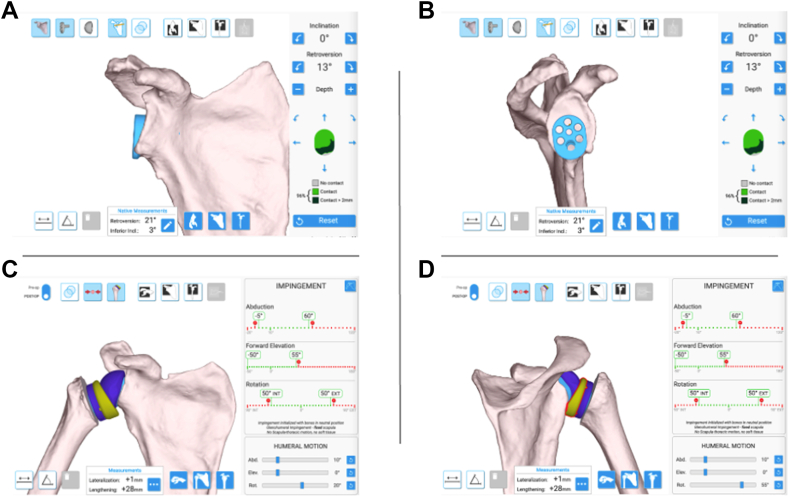
Preoperative planning with the Equinox Planning App allows surgeons to select component type and positioning relative to patient's three-dimensional generated native anatomy. (**A** and **B**) provide coronal and sagittal views, respectively, allowing the surgeon to adjust inclination and retroversion. (**C** and **D**) display glenosphere size and positioning in relation to the humeral component with calculation displaying expected range of motion in abduction, forward elevation, and rotation. The software is also capable of alerting surgeons to implant designs that may have higher risks of complications such as impingement illustrated in (**D**).

This is a retrospective comparative study for patients who underwent rTSA by a single surgeon (P.N.C.) from November 2022 to May 2025. The study included the last 84 consecutive primary, non-navigated surgeries and the subsequent 82 consecutive navigated surgeries from a single surgeon. During this period of time, this surgeon did not use patient-specific guides or bone grafting, but all cases, including those performed without navigation, utilized the Equinox Planning App for pre-operative computed tomography based planning regardless of implant type or manufacturer (Fig. 1). The surgeon made a wholesale change in their practice—ie, all patients prior to the switch were non-navigated and all patients after the switch were navigated, without any specific selection of patients for either group. This decision to include all implant manufacturers used during this transition was for the study to reflect a real-world transition other surgeons may face rather than an implant-specific comparison. The only exceptions were patients who were unable to obtain a pre-operative computed tomography scan and thus unable to undergo navigation. Institutional review board approval from the University of Utah was obtained before initiation of the study (IRB #71740).

A query of our electronic medical record was performed to identify patients with Current Procedural Terminology codes 23472 and 20985 for total shoulder arthroplasty and computer-assisted musculoskeletal surgical procedures, respectively. These codes were used to identify and create the non-navigation and navigation groups for the study. Patients in the consecutive series of 166 total patients were included regardless of indication. However, patients undergoing revision arthroplasty were excluded, as navigation could not be applied to revisions.

### Measurement technique

To evaluate radiographic differences between groups, a total of 4 measurements were performed for each patient: pre-operative and post-operative β-angle, inferior glenosphere offset, and the distance from the center of rotation of the glenosphere to the tip of the coracoid (COR-C).^5^^,^^14^^,^^18^^,^^25^ The pre-operative β-angle of the native glenoid is measured to define the inclination of the glenoid, where a larger β-angle represents a more superior tilt of the glenoid face.^25^

On plain radiographs, we made the following measurements using the IntelliSpace PACS system (Philips Healthcare, Amsterdam, Netherlands):

1. Pre-operative (Fig. 2*A*) and post-operative (Fig. 2*B*) inclination were measured using the β-angle on true anteroposterior views as previously described.^18^^,^^25^

**Figure 2:**
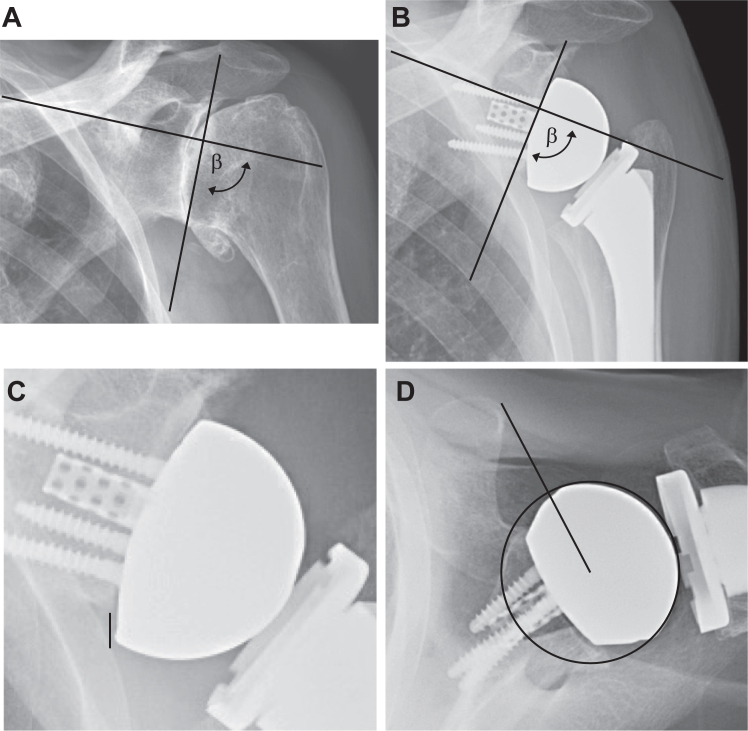
The measurement methods for (**A**) pre-operative inclination (β-angle), (**B**) post-operative β-angle, (**C**) inferior offset, and (**D**) center of rotation to the tip of the coracoid are demonstrated. Inclination, β-angle, and inferior offset were measured in true anteroposterior (Grashey) views. Center of rotation to the tip of the coracoid was measured with axillary views.
2. Inferior offset (glenoid component overhang) as the vertical distance between the inferior margin of the native glenoid and the most inferior point of the glenosphere (Fig. 2*C*).^5^
3. Center of rotation to the tip of the coracoid was the distance (mm) from the center of rotation to the posterior aspect of the coracoid on axillary radiographs as previously described (Fig. 2*D*).^14^

### Statistical methods

All analyses were performed in Excel X (Microsoft, Redmond, WA) and SPSS 25 (IBM, Armonk, NY, USA). To demonstrate measurement reliability, 50 radiographs were randomly selected and remeasured for each of the 4 radiographic parameters by a single author (R.A.A.) 2 weeks after initial measurements were performed. Intraclass correlation coefficients were obtained using a 2-way mixed-effects model for absolute agreement. To determine the association between navigation use and radiographic outcomes, we compared each of the radiographic measurements between the non-navigation and navigation groups using *t*-tests and Mann-Whitney U tests as appropriate depending upon data normality as determined using the Kolmogorov-Smirnov test. Categorical variables were compared using the Fisher–Freeman–Halton exact test for the comparison of implant type across navigated and non-navigated rTSAs.

An a priori power analysis was conducted using GPower (version 3.1.9.7; Universität Düsseldorf, Düsseldorf, Germany). Inclination was used as the primary outcome variable based upon a prior study in which inclination was demonstrated to associate with post-operative instability.^25^ Within that analysis, the unstable group had an inclination of 81, and the stable group had an inclination of 88 with a standard deviation of 10 in the unstable group. Based upon this magnitude of difference, with alpha set to 0.05, equal group sizes, 2-tailed tests, and without assuming normally distributed data, we needed 63 patients per group (126 patients total) to have a 95% chance of finding a difference should one exist. *P* values of <0.05 were considered statistically significant.

## Results

### Included patients, accuracy, and reliability

A total of 166 consecutive patients were included consisting of 84 non-navigated rTSAs followed by 82 rTSAs with computer-assisted navigation, all of which were performed by a single surgeon at a single institution. Between the 2 groups, there was a near even gender distribution between non-navigation (42 men, 42 women) and navigation (40 men, 42 women) groups (*P* = .875). There were no significant differences between the groups regarding age (*P* = .265), body mass index (*P* = .807), or surgical indications (*P* = .236; Table I). There was a significant difference in the Charlson Comorbidity Index with the non-navigated group having a lower score than the navigated group (3.8 ± 1.9 vs. 4.6 ± 2, *P* = .006) (Table II). Measurement reliability for radiographic measurements was found to be excellent with all intraclass correlation coefficients >0.799 (Table III).

**Table I:** Primary diagnoses among patients undergoing reverse total shoulder arthroplasty in the non-navigated (N = 84) and navigated (N = 82) groups.

Diagnosis	Non-navigation	Navigation	Total
GHOA with rotator cuff insufficiency	45 (53.6%)	44 (53.7%)	89 (53.6%)
Massive rotator cuff tear/failed rotator cuff repair	22 (26.2%)	20 (24.4%)	41 (25.3%)
Rotator cuff tear arthropathy	10 (11.9%)	16 (19.5%)	26 (15.7%)
Other∗	7 (8.3%)	2 (2.4%)	9 (5.4%)

*GHOA*, glenohumeral osteoarthritis.

∗ Other includes failed instability repair, chronic instability/dislocation, complication of prior surgery (hardware), inflammatory arthritis, and proximal humerus fracture.

**Table II:** Mean ± standard deviation for each measurement in both the non-navigation group (N = 84) and the navigation group (N = 82), as well as the mean difference between groups and 95% confidence intervals for this difference.

Variable	Non-navigation	Navigation	Mean difference [95% CI]	*P* value
Age	68.5 ± 7.7	69.9 ± 8	1.4 [−1.03 to 3.83]	.265
BMI	31.1 ± 6.7	31.4 ± 7.5	0.3 [−1.90 to 2.50]	.807
CCI	3.8 ± 1.9	4.6 ± 2	0.8 [0.20 to 1.40]	**.006**
Pre-operative inclination (°)	80.6 ± 7.7	82.9 ± 8.2	2.3 [−0.16 to 4.76]	.05
Post-operative inclination (°)	85.6 ± 7.7	86 ± 3	0.4 [−1.40 to 2.20]	.518
Inferior offset (mm)	5.4 ± 3.1	7 ± 2.5	1.6 [0.73 to 2.47]	**.001**
Center of rotation to coracoid (mm)	52.7 ± 9.5	52.8 ± 2.5	0.1 [−2.03 to 2.23]	.907
Operative time (min)	136 ± 30.8	141 ± 23.5	5 [−3.45 to 13.45]	**.031**
Pre-operative SSV	35.8 ± 19	40 ± 21.9	4.2 [−2.14 to 10.54]	.214
Pre-operative VAS	6.7 ± 2.1	6.3 ± 2.2	−0.4 [−1.06 to 0.26]	.187
Pre-operative ASES	34.8 ± 17	36.1 ± 17	1.3 [−3.95 to 6.55]	.565

*BMI*, body mass index; *CCI*, Charlson Comorbidity Index; *SSV*, subjective shoulder value; *VAS*, visual analog scale; *ASES*, American Shoulder and Elbow Surgeons; *CI*, confidence interval.

Significant differences are bolded.

**Table III:** Reliability statistics for each of the included variables.

Radiographs	Reliability ICC [95% CI]
Pre-operative inclination (°)	0.867 [0.745 to 0.931]
Post-operative inclination (°)	0.822 [0.7 to 0.898]
Inferior offset (mm)	0.855 [0.754 to 0.917]
Distance from center of rotation to coracoid (mm)	0.799 [0.65 to 0.886]

*ICC*, intraclass correlation coefficient; *CI*, confidence interval.

A significant association was observed between implant type and navigation status (*P* < .001). Non-navigated cases most commonly used the Exactech implant (67.9%), followed by DePuy (25.0%) and Tornier (7.1%), whereas all navigated cases exclusively used the Exactech implant (Table IV).

**Table IV:** Demographic and radiographic characteristics among patients undergoing reverse total shoulder arthroplasty among the 3 different implant systems used during the study period (DePuy, Exactech, and Tornier).

Variable	DePuy (n = 21)	Exactech (n = 139)	Tornier (n = 5)	*P* value
Age (yr, mean ± SD)	68.6 ± 8.3	69.1 ± 7.9	73.6 ± 4.5	.428
BMI (kg/m^2^, mean ± SD)	31 ± 7.9	31.4 ± 7	31.4 ± 9.3	.735
CCI (mean ± SD)	3.7 ± 1.9	4.3 ± 2	5.4 ± 1.5	.109
Pre-operative inclination (°, mean ± SD)	55.7 ± 38	69.3 ± 33	63.4 ± 40.9	.22
Post-operative inclination (°, mean ± SD)	81.6 ± 21.2	83.1 ± 16.3	85.3 ± 4	.899
Inferior offset (mm, mean ± SD)	3.4 ± 4.9	6.2 ± 3.7	5.2 ± 2.3	**.006**
Center of rotation to coracoid (mm, mean ± SD)	54 ± 9.3	50.6 ± 14.9	46.9 ± 5.8	.328

*BMI*, body mass index; *CCI*, Charlson Comorbidity Index; *SD*, standard deviation.

Significant differences are bolded.

### Radiographic outcomes

Inferior offset was significantly greater in the navigated group (7.0 ± 2.5 mm) compared to the non-navigated group (5.4 ± 3.1 mm; *P* = .001). Pre-operative inclination was not significantly different comparing groups (82.9 ± 8.2° vs. 80.6 ± 7.7°; *P* = .050). Post-operative inclinations (86.0 ± 3.0° vs. 85.6 ± 7.7°; *P* = .518) and center of rotation to coracoid distance (52.8 ± 2.5 mm vs. 52.7 ± 9.5 mm; *P* = .907) were not significantly different between the groups (Table I).

**Figure 3:**
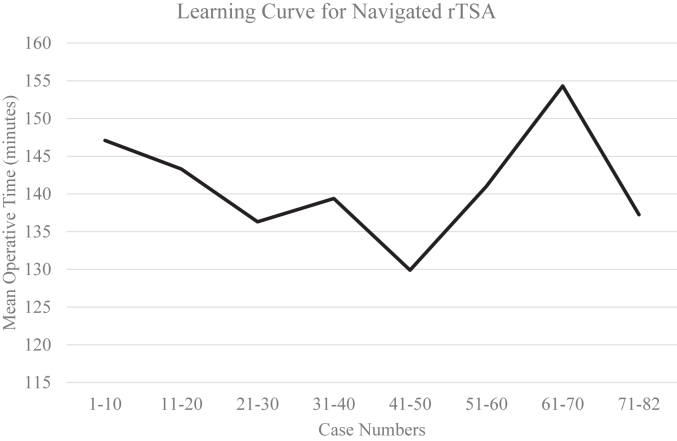
Mean operative time (minutes) is shown by sequential blocks of 10 cases with the final block containing 12 cases for a total of 82 navigated rTSA. *rTSA*, reverse total shoulder arthroplasty.

Mean operative time determined by anesthesia start and stop times was significantly higher in the navigated group (141 ± 23.5 minutes; *P* = .031) compared to the non-navigated group (136 ± 30.8 minutes, Fig. 3).

### Complications

Post-operative complications were recorded for all patients, with the non-navigated group having a longer mean follow-up duration, having been performed earlier in the surgeon's practice. The navigated group had a minimum of 3 months of follow-up at the time of the review.

When comparing the first 3 postoperative months, the period for which follow-up was available for all patients, the non-navigated group experienced 4 dislocations, compared with zero dislocations in the navigated group (*P* = .045).

Across the entire available follow-up period, additional complications in the non-navigated cohort included axillary nerve palsy (n = 1), baseplate failure (n = 1), fracture (n = 1), infection (n = 2), persistent pain (n = 1), and conjoint tendonitis (n = 2, including one requiring surgical release). In the navigated cohort, complications included suspected plexopathy (n = 3), acromial fracture (n = 3), conjoint tendonitis (n = 1), coracoid fracture (n = 1), infection (n = 2), persistent pain (n = 1), and strap tendonitis (n = 1). No cases of scapular notching or baseplate loosening were identified in either group during the available follow-up.

While the overall frequency of complications was similar between groups, their nature differed—mechanical complications (dislocation, baseplate failure) occurred exclusively in the non-navigated cohort, whereas stress-related events (acromial or coracoid fractures, plexopathy, and tendonitis) were observed primarily in the navigated cohort.

### Learning curve

**Figure 4:**
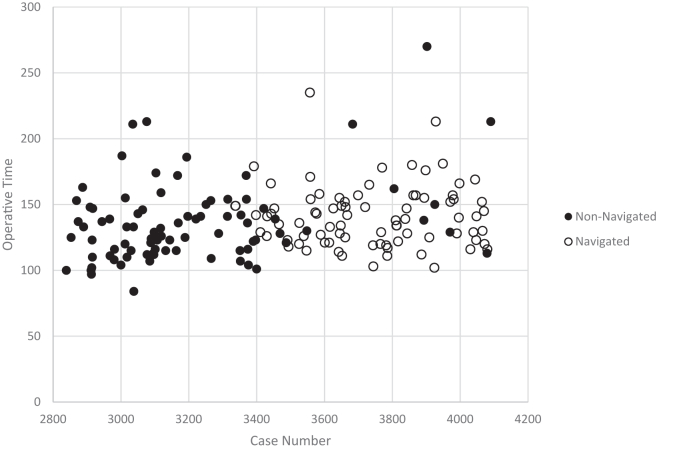
Scatterplot of operative time across sequential case numbers for reverse total shoulder arthroplasty. Each dot represents one case.

Operative time was evaluated sequentially across all 82 navigated rTSA cases to assess for a learning-curve effect. Linear regression analysis demonstrated no significant association between case order and operative time (*P* = .93) (Fig. 4).

## Discussion

This retrospective study evaluated the wholesale transition from non-navigated, or conventional, rTSA for a single surgeon transitioning to computer-assisted navigated rTSA with the goal of understanding the associated radiographic accuracy, early complications, and associated learning curve during this transition. We found that navigation increased operative time by 5 minutes but was associated with greater inferior glenoid component offset and a fewer events of early post-operative instability.^17^ These results provide unique insight for surgeons contemplating the transition to navigation as this tool for surgeons becomes more readily available and accessible.

Accurate glenoid component positioning optimizes impingement-free range of motion and implant survival. Instability is recognized as among the most common complications following rTSA.^4^^,^^6^^,^^7^^,^^19^^,^^20^^,^^25^ In this study, all cases were planned with an intended inferior offset of 6 mm based on pre-operative planning. The navigated group achieved a mean offset of 7.00 ± 3.1 mm compared with 5.4 ± 2.5 in the non-navigated group. Although both groups approximated the pre-operative plan with this respect, the narrower standard deviation in the navigation group suggests greater precision and reproducibility in achieving the desired offset. While we cannot conclude that navigation was more accurate than non-navigation, these findings indicate that navigation may improve consistency in replicating the pre-operative plan.^17^

All patients had at least a 3-month follow-up. During this immediate post-operative period, it was notable that the navigation group did not experience any dislocations. Though we cannot definitively conclude the reason for this dramatic improvement, the increased inferior offset may explain this improvement. Increased inferior offset reduces adduction impingement, which can contribute to instability.^16^ Despite these encouraging results, it remains uncertain whether the improved radiographic outcomes observed with navigation will directly translate into long-term implant survival or functional gains. Holzgrefe et al reported lower revision and complication rates in their navigation group, though their differences did not reach statistical significance.^12^ Therefore, longer-term follow-up and larger series are needed to confirm whether navigation ultimately reduces revision rates and improves patient-reported outcomes.

Despite the sudden integration of a new surgical technique with new technology and altered workflow, there was no significant difference in operative time from the first navigated case to the last one observed during the study period. The absence of a significant difference from case 1 to case 82 may indicate a rapid achievement of efficiency during this adoption process. In a recent systematic review, Menendez et al reported that surgeons typically require approximately 25 cases to achieve proficiency for non-navigated rTSA before experiencing reductions in operative time.^2^ The findings from this study suggest that navigation may mitigate this traditional learning effect, though further studies are necessary in order to investigate the learning curve effects of navigation on long-term complication rates and patient-reported outcomes.

With the adoption of new technology in the operating room, there are frequently many concerns including cost, space, and drawbacks during the transition period such as increased operative time and complication rates as surgeons become accustomed to a newer technique that may differ from one that had previously relied upon in their practice. This study, like many others, did note an increase in operative time by 5 minutes. Prior studies in the primary total hip arthroplasty have shown that a 10-minute increase in operative time may increase the risk of surgical site infections by approximately 7%.^24^ It remains unclear how impactful an addition of 5 minutes may be regarding complications such as surgical site infection, wound dehiscence, thromboembolism, or other complications related to operative time, especially as it pertains to shoulder arthroplasty. Surgeons will have to decide whether navigation provides enough benefit to justify this additional surgical time. It is the opinion of the senior author that this modest increase in surgical time is well justified by the decrease in post-operative instability and the likely additional associated decrease in associated post-operative complications such as scapular notching.^9^^,^^11^^,^^22^ However, other surgeons will have to decide whether the additional 5 minutes of anesthesia time is a reasonable trade-off for their practice.

## Limitations

This study does contain limitations. Due to the nature of this retrospective, non-randomized study, there remains the potential for a selection bias among patients included. The use of a single surgeon who underwent a wholesale, consecutive transition in their practice from non-navigation to navigation was an attempt to strengthen the internal validity. This also provided a consistent set of indications and allows direct comparison between the 2 techniques, associated learning curve, and minimizes confounding variables introduced with the inclusion of other surgeons or institutions. However, this also limits the generalizability of these findings across a broader practice setting. Additionally, the surgeon's practice and techniques remained stable during the study period, meaning that outside of the introduction of navigation to their practice, other factors during the patient's workup and surgical management were consistent and most comparable. However, certainly subtle changes are possible with time, in particular with implant selection. The short-term follow-up limits our ability to draw conclusions about the effect of navigation upon implant longevity or patient outcomes. The small sample size may be underpowered for some comparisons, but an a priori power analysis shows our analysis to be well powered for inclination.

Another limitation is that implant types differed significantly between the non-navigated and navigated groups as the surgeon transitioned implant systems during the study period. Because navigation was introduced during a period in which a transition was also made to a new implant system, it is difficult to determine whether the differences observed between groups are due to the use of navigation itself, the implant design, or combination thereof.

The use of IntelliSpace PACS system may also introduce limitations in this study regarding the accuracy and precision of the measurements obtained. As far as the authors understand, IntelliSpace does not quantify an intrinsic error value to report. Additionally, the use of radiographic measurements may be less accurate than if the same measurements had been performed using computed tomography. We believe that by using a validated imaging platform, standardizing measuring techniques based on prior published studies, and obtaining excellent reliability, we were able to mitigate any intrinsic error present in the IntelliSpace PACS system.

This study also remains limited due to the absence of patient-reported outcomes. Due to the nature of the short follow-up in the navigated group, it is difficult to assess how impactful improved implant accuracy with the navigation group will affect patient outcomes. This study's conclusions are primarily focused on the technical aspects of implant positioning, which represent just one of many factors that may impact patient outcomes. Other considerations for patient's improvement include soft tissue balance and patient-specific anatomy.

## Conclusion

This retrospective single-surgeon study illustrates that transition to computer-assisted navigation in rTSA may modestly increase operative time while improving implant positioning including inferior glenoid offset. No early postoperative dislocations were observed in the navigated group, though definitive conclusions are limited by short follow-up and limited sample size. This study demonstrates an association between the role of computer-assisted navigation in improving implant accuracy, but further studies are needed to determine its clinical relevance.

## Disclaimers:

Funding: No funding was disclosed by the authors.

Conflicts of interest: Christopher Joyce is a paid consultant for Zimmer Biomet.

Robert Tashjian is a paid consultant for Zimmer Biomet, Enovis, and Stryker; has stock in Conextions and Genesis; receives intellectual property (IP) royalites from Zimmer Biomet, Stryker, and Shoulder Innovations; receives publishing royalties from Spring and the *Journal of Bone and Joint Surgery*; and serves on the editorial board of the *Journal of Bone and Joint Surgery*.

Peter Chalmers is a paid consultant for DePuy, Smith+Nephew, Responsive Arthroscopy, and Exactech, receives IP royalites from DePuy, Responsive Arthroscopy, and Exactech; has stock in TitinKM Biomedical; receives research support from DePuy, Smith+Nephew, and the National Institutes of Health; and serves on the editorial board of the *Journal of Shoulder and Elbow Surgery* and serves as the Editor-in-Chief for the *Journal of Shoulder and Elbow Surgery*: *Reviews, Reports, and Techniques*.

Any additional authors, their immediate families, and any research foundations with which they are affiliated have not received any financial payments or other benefits from any commercial entity related to the subject of this article.

Given his role as Editor in Chief, Dr. Peter N. Chalmers had no involvement in the peer-review of this article and has no access to information regarding its peer-review. Full responsibility for the editorial process for this article was delegated to Dr. John W. Sperling.
